# More miles on the clock: Neighbourhood stressors are associated with telomere length in a longitudinal study

**DOI:** 10.1371/journal.pone.0214380

**Published:** 2019-03-28

**Authors:** Anne Ellaway, Ruth Dundas, Tony Robertson, Paul G. Shiels

**Affiliations:** 1 MRC/CSO Social and Public Health Sciences Unit, University of Glasgow, Glasgow, United Kingdom; 2 Centre for Public Health and Population Health Research, Faculty of Health Sciences & Sport, University of Stirling, Stirling, United Kingdom; 3 Institute of Cancer Sciences, University of Glasgow, Glasgow, United Kingdom; University of Maiduguri College of Medical Sciences, NIGERIA

## Abstract

**Background:**

There is a substantial gap in health and longevity between more affluent and more deprived areas, and more knowledge of the determinants of this health divide is required. Experience of the local residential environment is important for health although few studies have examined this in relation to biological markers of age such as telomere length. We sought to examine if residents’ perceptions of neighbourhood stressors over time were associated with telomere length in a community study.

**Methodology/Principal findings:**

In a prospective cohort study of 2186 adults in the West of Scotland, we measured neighbourhood stressors at three time points over a 12-year period and telomere length at the end of the study. Using linear regression models, we found that a higher accumulation of neighbourhood stressors over time was associated with shorter telomere length, even after taking cohort, social class, health behaviours (smoking status, diet, physical activity), BMI and depression into account among females only (Beta = 0.007; 95%CI [0.001, 0.012]; P<0.014).

**Conclusions/Significance:**

Neighborhood environments are potentially modifiable, and future efforts directed towards improving deleterious local environments may be useful to lessen telomere attrition.

## Introduction

A number of studies have shown that residents’ perceptions of the local environment are associated with their health, independently of individual characteristics such as socioeconomic status (SES), age and gender [1–5]. However, the majority of the findings to date are based on self-reported health outcomes, leaving open the possibility of ‘plaintive-set’ reporting bias [6]. Moreover, given the substantial gap in health and longevity between more affluent and more deprived areas [7, 8], more knowledge on the determining factors of poorer health in deprived areas, particularly in relation to biological mechanisms over the life course [9] are required to inform interventions [10]. It has been suggested that neighbourhood perceptions are important to examine as they can stimulate psychological and/or physiological stress responses that can affect mental and physical health [11]. One measure, which has received increased attention in relation to life stress and accelerated aging, is telomere shortening [12, 13].

Telomeres are specialised nucleoprotein complexes at the end of chromosomes which maintain genomic integrity by providing a cap for the chromosome, thus preventing chromosomal fusions [14]. Telomeres typically shorten during somatic cell division as a consequence of the ‘end replication problem’ [15, 16]. Telomere length has been posited as good marker for aging [17] and shorter telomeres have been observed by individual characteristics such as sex, among males compared to females [18] and lower SES in some populations [13, 19] but not others [20]. Shortened telomere length has been associated with exposure to chronic psychological stress [21], depression [22] and a range of non-communicable diseases including cancer [23] chronic kidney disease and diabetes [24]. As telomere length is modifiable [25] [23], a more nuanced understanding of the underlying mechanisms which facilitate accelerated telomere shortening can provide useful information to inform potential interventions that may mitigate the rate of telomeric attrition [10].

To date, only a small number of studies have examined whether exposure to neighbourhood conditions are associated with telomere length. In the USA, shorter telomeres were associated with perceived neighbourhood safety and social cohesion in the Multi-Ethnic Study of Atherosclerosis (MESA) [26]; perceived neighbourhood problems such as heavy traffic and excessive noise in a sample of African American females [27]; and in the Netherlands, perceived neighbourhood disorder (vandalism), fear of crime and noise were associated with telomere shortening [11]. As we have previously shown that social housing tenure is associated with shorter telomere length [28], and given that social housing tenure may expose individuals to poorer neighbourhood quality [29] it is key to explore whether neighbourhood quality is part of the pathway linking housing tenure to telomere attrition. Moreover, as most studies to date which have examined perceptions of the neighbourhood and telomere length are cross sectional, there have been calls to examine the effects of cumulative exposures to adverse neighbourhood conditions on health [30]. As others have discussed [10, 11, 26, 31], prolonged exposure to stressful conditions may lead to chronic activation of the hypothalamic–pituitary–adrenal (HPA) axis, which may influence the rate of telomere shortening, through changes in hallmark processes of ageing [32, 33]. Indirect mechanisms whereby chronic stress may invoke telomere shortening are related to the exposome [34] and include unhealthy behaviours as coping strategies such as the consumption of ‘comfort’ foods or smoking to alleviate stress. In this study, we have examined whether perceived neighbourhood stressors were associated with telomere length using three sweeps of data (hereby referred to as ‘waves’), covering 13 years of the life course, from three cohorts in the longitudinal West of Scotland Twenty-07 Study. We therefore assessed two models [35] to examine the impact of perceived neighbourhood stressors on telomere length. These comprised: (i) an accumulation model which uses a cumulative measure of neighbourhood perceptions over time; (ii) a critical periods model which separately analyses the relationship between measures of perceived neighbourhood stressors at each wave and subsequent telomere length. As a number of studies have shown that neighbourhood conditions may impact differently upon the health of males and females [1, 36–39] we conduct our analyses separately by sex.

## Methods

Data were from the West of Scotland Twenty-07 Study, a community-based, prospective cohort study, which has followed three cohorts of men and women recruited (one individual per household) in 1987 at the (approximate) ages of 15 (‘1970s cohort’), 35 (‘1950s cohort’) and 55 years (‘1930s cohort’) in 1987 (wave 1) and the same individuals were followed up in a further four waves over the next 20 years until 2007/8. Baseline respondents have been shown to be representative of the general population of the sampled area [40]. At Wave 5, 83.6% of those eligible (i.e. still living and contactable) to take part participated in an interview. As expected in a general population longitudinal survey, men, those in the oldest cohort and people from manual occupations were more likely to have died. The study design is described in more detail elsewhere [41]. The Tayside Committee on Medical Research Ethics approved the study. Informed, written consent was obtained from all respondents at each wave of the study. Here, we use three waves of the study for which we have data relevant to our research question in this paper (wave 3, 4 & 5). Data, including blood samples at wave 5 (2007/8, n = 2604), were collected by trained nurses in the homes of the study participants when respondents were aged approximately 35 (1970s cohort), 55 (1950s cohort) and 75 years (1930s cohort).

### Individual level variables

Household social class (an accepted measure of socioeconomic position [42]) was coded into six classifications I- VI (I Professional, II Managerial and Technical, III skilled non manual, IV skilled Manual, IV partly skilled occupations, VI unskilled occupation) according to the Registrar General’s 1980 classification [43] for head of household's current or previous occupation. Depression, which was added as a covariate in models to partly account for the potential of poor mental health influencing perceptions of the local environment, and as it has been shown to be related to telomere length [22], was measured by the 7 item depression subscale of the Hospital Anxiety and Depression Scale [HADS], higher scores on HADS indicate greater reported symptoms. [44]. As this instrument was not available for all respondents who participated at Wave 3, a single item (‘feeling unhappy or depressed over the previous week’) from the 12 item General Health Questionnaire (GHQ-12 [45]) was used instead for critical period (cross sectional). Health behaviours, as correlates of telomere length, were assessed at Wave 5, include diet (consumption of sweets and chocolate) [46], smoking status [47], physical activity (‘In an average week, on how many days do you spend at least 20 continuous minutes doing vigorous physical exercise, enough to make you sweaty and out of breath?’) [48] and Body Mass Index (BMI: weight [kg]/height[m]^2^)[49].

### Biospecimen collection procedure

At Wave 5, respondents’ blood was drawn by venepuncture from the median cubital vein (major arm vein) at the end of the interview. Whole blood (4 ml) was collected in potassium ethylenediaminetetraacetic acid vacutainer tubes (Becton Dickinson, Franklin Lakes, NJ, USA). The tubes were stored at 4-C until DNA extraction. DNA was extracted from peripheral blood leukocytes using the Maxwell automated purification system, according to the manufacturer’s instructions (Promega, Fitchburg, WI, USA). The DNA concentration and purity were quantified using the Nanodrop spectrophotometer (Thermo Fisher Scientific, Waltham, MA, USA).

### Telomere length

As previously reported [20, 22, 28], telomere length was analysed at the Institute of Cancer Sciences, University of Glasgow. Telomere lengths in the DNA extracted from peripheral blood leukocytes were determined by quantitative polymerase chain reaction, following the method of Cawthon [50]. Telomere length determination was performed blindly using a Roche Light Cycler LC480. Telomere length analyses were performed in triplicate for each sample, using a single-copy gene amplicon primer set (acidic ribosomal phosphoprotein, 36B4) and a telomere-specific amplicon primer set [51]. Quality control parameters used for the amplifications comprised a cut-off of 0.15 for the standard deviation (SD) of the threshold cycle (Ct) for sample replicates. At an SD above 0.15, the sample was reanalyzed. The average SD across plates was 0.05. A control DNA sample was used to generate a standard curve on every plate to enable absolute quantification analysis. Relative telomere length was estimated from Ct scores using the comparative Ct method after confirming that the telomere and control gene assays yielded similar amplification efficiencies. This method determines the ratio of telomere repeat-copy number to single-copy gene number (T/S) in experimental samples relative to a control sample DNA. This normalized T/S ratio was used as the estimate of relative telomere length (relative T/S). It should be noted that the same control DNA sample was used on every plate that was analyzed. The intra-assay variation was assessed by comparing the relative telomere estimates (T/S ratio) across assays for the positive controls, which were assayed on every assay plate. The average intra-assay coefficient of variance was 0.56% for telomere and 0.19% for 36B4 plates [13, 50, 52].

### Perceptions of neighbourhood quality

A similar suite of questions on respondents’ perceptions of their local neighbourhood was asked at three waves of the study: wave 3 (1995), wave 4 (2000) and wave 5 (2007). Respondents were asked ‘Around where you live would you say that any of the following are a serious problem, a minor problem or not a problem’ and provided with a list of six socio-environmental problems: vandalism, litter and rubbish, assaults and muggings, disturbances by children or youngsters, smells and fumes, burglaries and invited to reply using a three-point scale ('not a problem' score 1, 'minor problem' score 2, 'serious problem' score 3). A similar suite of questions has been used in other UK studies including the General Household Survey and the Hereford Cohort Study [53, 54]. At each wave, to provide a more differentiated score on the perceived severity of the problems [55, 56], scores were revised so that ‘serious problem’ was given a score of one and ‘minor problem’ and ‘not a problem’ were each scored as zero. Responses to each of the individual problems were then summed, with scores ranging from 0 to 6 at each wave, with the higher score indicating greater problems in the neighbourhood. To create a cumulative measure of neighbourhood perceptions over time, scores were summed across the three waves and scores for this variable ranged from 0 to 15. All neighbourhood perceptions scores were measured and analysed at the individual level.

### Statistical analysis

Associations between T/S (relative telomere length) and two models (‘accumulation’, critical’) of perceptions of the neighbourhood were analyzed, separately for males and females, using linear regression models, adjusted for cohort, household social class, the HADS measure of depression, diet [46], smoking status [47], physical [48] and Body Mass Index [49]. All analyses were performed with IBM SPSS 21.

## Results

Of the 2604 study participants who took part at wave 5, 2310 (88.7%) consented to have telomere analysis performed; 2193 (94.9%) participants had sufficient blood for DNA to be extracted. Telomere length was successfully measured for 2186 (94.6%) (1970s cohort n = 776; 1950s cohort n = 866; 1930s cohort n = 544). There were various reasons for non-collection of blood among those who had consented, for example: blood not running freely enough to permit collection (n = 53), the respondent being pregnant at the time of the interview (n = 11), respondent feeling unwell (n = 17), sample only partially collected (n = 79).

Table 1 provides descriptive statistics for the Wave 5 sample broken down by sex, age cohort and social class. Just over half of the sample (54.4%, n = 1192) were female, 35.4% were aged around 35, 39.7% aged around 55 and 25% aged around 75. The mean T/S for the sample was 0.79 (SD 0.21), males had shorter telomeres than females, telomere length was longest among the youngest cohort and shorter among lower social class groups. The mean (SD) for neighbourhood stressors as measured over 3 waves (the accumulation measure) was 1.24 (SD 0.21); for wave 3 this was 0.46 (SD 1.00); at wave 4 it was 0.37 (SD 0.89) and 0.441 at wave 5 (0.96). Females had higher scores for most measures of neighbourhood stressors (except wave 4), but this was not statistically significant. The oldest cohort had higher scores in the main compared to the younger cohorts, although this was only statistically significant at wave 4. For the accumulation score and for all 3 cross sectional waves, significantly higher scores for neighbourhood problems were observed among lower social class groups (p<0.001).

**Table 1:** Mean telomere length by sex, age cohort and social class.

	n	%	T/S ratio Mean (SD), p<	Accumulation of neighbourhood problems *mean (SD)*	Neighbourhood problems Wave 3 *mean (SD)*	Neighbourhood problems Wave 4 *mean (SD)*	Neighbourhood problems Wave 5 *mean (SD)*
**sex**			P< 0.021				
male	994	45.6	0.78 (0.21)	1.16 (2.07)	0.43 (0.96)	0.36 (0.91)	0.38 (0.94)
females	1192	54.4	0.80 (0.20)	1.31 (2.14)	0.49 (1.03)	0.38 (0.86)	0.44 (0.98)
**cohort**			P< 0.001			P<0.020	
1970s	776	35.4	0.86 (0.21)	1.20 (1.96)	0.48 (1.05)	0.33 (0.86)	0.40 (0.92)
1950s	866	39.7	0.78 (0.19)	1.20 (2.15)	0.44 (0.98)	0.35 (0.84)	0.42 (0.98)
1930s	544	25.0	0.70 (0.19)	1.37 (2.24)	0.48 (0.96)	0.46 (0.99)	0.43 (1.00)
**Head of Household social class**			P< 0.001	P< 0.001	P< 0.001	P< 0.001	P< 0.001
I Professional	266		0.81 (0.21)	0.71 (1.27)	0.27 (0.67)	0.23 (0.61)	0.22 (0.60)
II managerial and technical	896		0.81 (0.21)	0.99 (1.81)	0.40 (0.94)	0.30 (0.77)	0.30 (0.77)
III skilled non manual	457		0.78 (0.19)	1.35 (2.13)	0.48 (0.98)	0.41 (0.89)	0.46 (1.04)
III skilled manual	300		0.77 (0.20)	1.58 (2.61)	0.60 (1.19)	0.40 (1.02)	0.60 (1.17)
IV partly skilled	199		0.75 (0.21)	2.08 (2.67)	0.67 (1.23)	0.64 (1.23)	0.75 (1.30)
V unskilled occupation	67		0.73 (0.23)	2.18 (2.63)	0.78 (1.14)	0.70 (1.13)	0.70 (1.24)
Total	2186	100	O.79 (0.21)	1.24 (2.11)	0.46 (1.00)	0.37(0.89)	0.41(0.96)

In models adjusted for age cohort, social class, depression, smoking status, diet, physical activity and BMI (Table 2), neighbourhood stressors were significantly associated with telomere in the accumulation model, with poorer neighbourhood perceptions resulting in shorter telomere length for women only (Beta = 0.007; 95%CI [0.001, 0.012]; P<0.014). Looking at critical periods, (i.e. for each wave separately), neighbourhood stressors reported at Wave 5 were associated with telomere length again among women only (Beta = 0.015; 95%CI [0.003, 0.027]; p<0.016).

**Table 2:** Associations between perceived neighbourhood stressors and telomere length.

Perceived neighbourhood problems	*n*	β	CI	*P<*
**Accumulation**				
***Neighbourhood problems summed across 3 waves***				
*males*	951	0.002	-0.005, 0.008	*0*.*636*
*females*	1143	0.007	0.001, 0.012	***0*.*014***
**Critical periods**				
***Wave 3***				
*males*	951	0.002	-0.012, 0.015	*0*.*826*
*females*	1143	0.010	-0.001, 0.021	*0*.*083*
***Wave 4***				
*males*	951	0.008	-0.006, 0.023	*0*.*249*
*females*				
***Wave 5***				
*males*	**951**	-0.002	-0.016, 0.012	*0*.*741*
*females*	1143	0.015	0.003, 0.027	***0*.*016***

## Discussion

In this study, perceived neighbourhood stressors are associated with biological aging as measured by telomere length, even after taking known correlates such as chronological age, SES/social class, mental health (depression), health behaviours (smoking, diet, physical activity) and BMI into account. Our study is novel and adds to the literature, as although there is a growing body of research on the influence of characteristics of the neighbourhood environment and aging [10, 11, 26, 27], most studies to date which have explored perceptions of the local neighbourhood with telomere length have been cross sectional and few have investigated sex differences in these. Our models have explored perceptions of the neighbourhood measured over a 13-year time period and shown that the accumulation of neighbourhood stressors over time, and contemporaneous conditions, impact on telomere length. We found few associations between neighbourhood perceptions measured at earlier waves being associated with telomere length when examined cross-sectionally.

We found a stronger relationship between neighbourhood perceptions and telomere length for women compared to men. Other studies have not explored sex differences in the relationship between perceptions of neighbourhood and telomere length [11, 26] or have focussed on only one sex [27]. The gendered experience and consequences of place for health has previously been reported. For example, in studies comparing men and women, perceived neighbourhood crime is more strongly associated with the likelihood of women being a smoker [38], and neighbourhood trust was more strongly related to self-rated health among women [39]. The reasons for this may include differential exposure (women may spend more time than men in their local neighbourhood); women may be more sensitised to the quality of their neighbourhood, perhaps due to their being more likely to be the main carers of children; it is also possible that men and women process stressful environments differently in ways which might lead to different physiological responses. For example, it has previously been noted that there are sex differences in activation of the HPA axis as a response to stressful conditions [57].

Our findings are robust with respect to a measure of mental health (depression) known to be associated with telomere length [58]. In addition, by including depression in our models, we have partly accounted for the possibility that more negative perceptions of the neighbourhood are merely reflecting depressive symptoms. Moreover, depression may result from chronic stress which impacts on body systems (such as the immune system) which can affect telomere length [21, 22]. We also controlled for other health behaviours known to be associated with telomere length such as smoking status, diet, physical activity and a measure of obesity (BMI).

Our study has been able to examine the associations between exposures of neighbourhood stressors over time in a large well characterized population study comprising a wide range of ages. Although not identical, our measures of perceived neighbourhood problems are broadly similar to other studies in the USA [26] and the Netherlands [11] which have explored the influence of these perceived problems on telomere length. A limitation of our study is that telomere length was only measured at one time point, the final wave. However, as we have previously noted, given that assays for this are a relatively recent development and the difficulties of collecting blood samples in large epidemiological studies, this is still one of the largest studies to examine telomere length across different age and SES groups [22]. The telomere length data used in our analyses were generated historically and contemporary with the original Cawthon method, prior to an updated methodology from his lab [59]. Our findings that perceived neighbourhood problems are associated with telomere length may be subject to residual confounding in that there are likely to be unmeasured socioeconomic circumstances that affect these associations, particularly those measured across the life course [60, 61]. Health selection may also play a part in that individuals in poor health may be ‘sorted’ into social housing (and associated poor neighbourhood conditions) due to the UK priority points system [60, 62]. In addition, we do not have data on how long respondents spend in their neighbourhoods on a day-to-day basis and therefore the extent of their exposure to neighbourhood conditions. Finally, although we have found that chronic environmental stressors, such as neighbourhood problems accumulated over time, are related to shortened telomere length, we do not have a measure of physiological stress in our study and hence are unable to examine direct biological pathways between neighbourhood conditions and telomere length.

In conclusion, we have shown that chronic neighbourhood stressors are associated with shorter telomere length, particularly among women. Neighborhood environments are potentially modifiable, and future efforts directed towards improving deleterious local environments may be useful to lessen telomere attrition.
